# Late-onset swallowing outcomes post-treatment for head and neck cancer in a UK-based population

**DOI:** 10.1017/S0022215122000834

**Published:** 2023-03

**Authors:** S Tengku, I Lohi, A Connelly, E Slaven, K Sloane, K Herity, L McBlain, C M Douglas, J Montgomery

**Affiliations:** 1 School of Medicine, University of Glasgow, Glasgow, Scotland, UK; 2 Department of Otolaryngology, Head and Neck Surgery, Queen Elizabeth University Hospital, Glasgow, Scotland, UK; 3 Department of Speech and Language Therapy, Queen Elizabeth University Hospital, Glasgow, Scotland, UK

**Keywords:** Head And Neck Neoplasms, Dysphagia, Swallowing, Radiotherapy, Quality Of Life

## Abstract

**Background:**

Adverse swallowing outcomes following head and neck squamous cell carcinoma treatment in the context of late-onset post-radiotherapy changes can occur more than five years post-treatment.

**Methods:**

A retrospective study was conducted utilising patient records from March 2013 to April 2015. Patients were categorised into ‘swallow dysfunction’ and ‘normal swallow’ groups. Quality of life was investigated using the MD Anderson Dysphagia Inventory and EuroQol questionnaires.

**Results:**

Swallow dysfunction was seen in 77 (51 per cent) of 152 patients. Twenty-eight patients (36 per cent) in the swallow dysfunction group reported symptoms in year five. Swallow dysfunction was associated with stage IV head and neck squamous cell carcinoma (*p* < 0.001) and radiotherapy (*p* < 0.001). MD Anderson Dysphagia Inventory global scores showed significant differences between swallow dysfunction and normal swallow groups (*p* = 0.01), and radiotherapy and surgery groups (*p* = 0.03), but there were no significant differences between these groups in terms of MD Anderson Dysphagia Inventory composite or EuroQol five-dimensions instrument scores.

**Conclusion:**

One-third of head and neck squamous cell carcinoma survivors with swallow dysfunction still show symptoms at more than five years post-surgery, a point at which they are typically discharged.

## Introduction

Head and neck cancers are a heterogeneous group of cancers emerging from the squamous epithelium of the head and neck. Risk factors include smoking, excess alcohol consumption and human papillomavirus (HPV) infection.^1^ Treatment modalities include surgery, radiotherapy (RT) and chemotherapy, or combinations thereof. These cancers and their treatments can have short- and long-term morbidity, impacting on quality of life (QoL).^2^

Head and neck cancer patients comprise 3 per cent of all cancer survivors.^3^ The overall five-year survival rate of head and neck cancer has improved from 54.7 per cent (during 1992–1996) to 65.9 per cent (during 2002–2006), and continues to rise because of the improved survivability of HPV-positive oropharyngeal cancer seen in younger patients.^4,5^ Long-term survivorship in head and neck cancer is an increasingly studied topic.^6^

Head and neck cancer treatments can lead to long-term morbidities, such as disfigurement, xerostomia, trismus, speech difficulties and dysphagia, including aspiration.^7^ The concept of ‘late radiation-associated dysphagia’ describes radiation-associated dysphagia that occurs years after treatment involving RT. Swallow dysfunction is a demanding late effect that may develop or progress years post-treatment. Post-RT neuromuscular fibrosis and stenosis may develop, resulting in an uncoordinated, inefficient and unsafe swallow, with long-term dysphagia.^8^ Dysphagia severity has been shown to be a strong predictor of survival; patients with very severe dysphagia and a ‘nil by mouth’ status have lower survival rates.^9^

Chronic dysphagia is strongly associated with reduced QoL and slower recovery time.^10^ A recent paper from Newcastle upon Tyne, which explored the late effects of organ preservation treatment on swallowing and voice, concluded that reliable and repeatable screening tools in the long-term follow up of head and neck cancer survivors are crucial for early recognition of at-risk patients.^11^

Swallowing outcomes five years post-treatment were studied in a US population of head and neck cancer survivors.^12^ This US case series from 2011 studied post-treatment outcomes in 29 patients previously treated with RT or chemotherapy. The study concluded that, despite functional preservation of organs being commonly obtained, late effects of RT were common, significant and difficult to treat.^12^

The current study aimed to identify and explore an equivalent head and neck cancer patient group within a UK population. We aimed to describe swallowing outcomes five years post-treatment and determine how these differ between primary treatment cohorts. Furthermore, we aimed to evaluate QoL data in relation to swallowing outcomes after five years of follow up, using validated questionnaires.

## Materials and methods

A review of National Health Service (NHS) Greater Glasgow and Clyde electronic patient records from March 2013 to April 2015 was performed. Patients were identified from a head and neck cancer multidisciplinary team database and selected according to pre-determined inclusion and exclusion criteria.

### Patients

All included patients had new diagnoses of head and neck squamous cell carcinoma (SCC) of an applicable site: hypopharynx, larynx, nasopharynx, neck with an unknown primary, oral cavity, oropharynx, salivary gland, or sinonasal or synchronous tumours. Only patients who received curative intent treatment, consisting of surgery, RT, chemotherapy, or a combination thereof, and who survived at least five years from the date of diagnosis, were included. Patients who did not survive five years, had recurrent cancer or did not have sufficient information on the NHS Clinical Portal were excluded.

The included patients were categorised into two groups: ‘swallow dysfunction’ and ‘normal swallow’ groups. Patients in the swallow dysfunction group showed at least one type of evidence of swallow dysfunction during the five years post-treatment, which could include self-reported or any objectively observed form of dysphagia, as shown in Table 1. Patients in the normal swallow group met the overall inclusion criteria, but not the swallow dysfunction criteria.

**Table 1.** Evidence of swallow dysfunction

Evidence	Patients (*n* (%))
Documentation of self &/or clinician reported dysphagia	77 (100)
≥1 Videofluoroscopy with aspiration &/or stricture	20 (26)
≥1 Barium swallow with aspiration &/or stricture	10 (13)
Requirement for oesophageal dilatation	9 (12)
≥1 Episode of hospitalisation with aspiration pneumonia	7 (9)
Evolving documented cranial nerve palsy	2 (3)

### Instruments

Two questionnaires were utilised: the MD Anderson Dysphagia Inventory and the EuroQol five-dimensions (‘EQ-5D’) instrument. The questionnaires were carried out via telephone calls as a result of restrictions during the height of the coronavirus disease 2019 pandemic.

The MD Anderson Dysphagia Inventory is a well-validated questionnaire for use in head and neck cancer patients. It consists of 20 items, each scored on a 5-point Likert scale, from which 2 summary scores are derived.^13^ The global score is a single question scored separately that assesses the perceived overall effect of swallowing abilities on QoL. The remaining 19 questions give a composite score ranging from 20 (indicating extremely low day-to-day functioning) to 100 (high day-to-day functioning).

The EuroQol five-dimensions instrument is a validated questionnaire consisting of a descriptive measure of health status (EuroQol five-dimensions five-levels; ‘EQ-5D-5L’) and a EuroQol visual analogue scale (VAS) (‘EQ-VAS’). The EuroQol VAS is a rating of overall health on a scale of 0 (worst imaginable health) to 100 (best imaginable health).^14^

### Statistical analysis

Descriptive statistics were calculated to describe patient demographics, disease characteristics and treatment outcomes. Statistical data were analysed using SPSS Statistics for Macintosh software, version 27.0 (IBM, Armonk, New York, USA). Chi-square tests and Fisher's exact tests were used to compare swallow dysfunction and normal swallow groups. Mann–Whitney U tests were used to compare MD Anderson Dysphagia Inventory and EuroQol VAS scores between these swallow groups, and between RT and surgery groups. Spearman's rho and Pearson's r were used to determine significant correlations between MD Anderson Dysphagia Inventory and EuroQol five-dimensions instrument scores. Results were considered statistically significant if *p* < 0.05.

### Ethical considerations

UK research ethics committee advice was sought using the online tool from the NHS Health Research Authority and Medical Research Council website; formal ethical review was not required.^15^

## Results

### Patient demographics

**Fig. 1.**
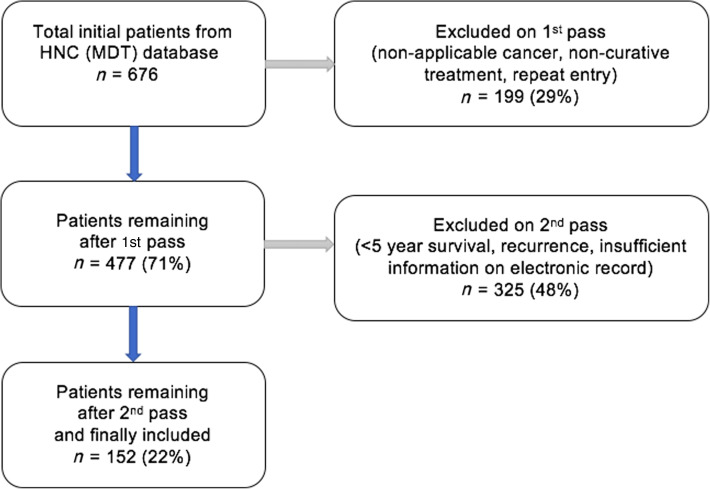
Numbers of patients included and excluded after applying pre-determined criteria. HNC = head and neck cancer; MDT = multidisciplinary team

Between March 2013 and April 2015, 676 patients were diagnosed with head and neck cancer. As shown in Figure 1, approximately one-third of these patients did not have cancer of an applicable site or did not receive curative treatment, and were therefore excluded. Another half were excluded because of: disease recurrence, less than five-year survival or insufficient documentation.

A total of 152 patients (22 per cent), who had a newly diagnosed head and neck SCC of an applicable site, underwent curative treatment, and reached five-year survival, were included. The mean follow-up time for all included patients was five years and six months (range, 5 years – 6 years and 10 months).

Table 2 shows the patient, tumour and treatment characteristics of all included patients at the time of diagnosis. Male gender (*n* = 103, 68 per cent), a history of smoking (*n* = 111, 73 per cent) and a body mass index above the healthy range (*n* = 96, 63 per cent) predominated in this patient cohort.

**Table 2.** Patient, tumour and treatment characteristics

Characteristic	All patients (*n* = 152)	Swallow dysfunction group (*n* = 77)	Normal swallow group (*n* = 75)	*P*-value
Sex (*n* (%))				
– Male	103 (68)	54 (70)	49 (65)	
– Female	49 (32)	23 (30)	26 (35)	
Age (years)				
– Mean	60	58	61	
– Range	19–87	19–80	36–87	
Alcohol consumption (n (%))				
– ≤14 units per week	105 (69)	54 (70)	51 (68)	
– >14 units per week	47 (31)	23 (30)	24 (32)	
Smoking status (*n* (%))				
– Current smoker	65 (43)	32 (42)	33 (44)	
– Ex-smoker	46 (30)	25 (32)	21 (28)	
– Never smoked	41 (27)	20 (26)	21 (28)	
Body mass index (*n* (%))				
– Underweight (<18.5 kg/m^2^)	11 (7)	4 (5)	7 (9)	
– Healthy (18.5–24.9 kg/m^2^)	43 (28)	17 (22)	26 (35)	
– Overweight (25–29.9 kg/m^2^)	65 (43)	38 (49)	27 (36)	
– Obese (≥30 kg/m^2^)	31 (20)	17 (22)	14 (19)	
– Unknown	2 (1)	1 (1)	1 (1)	
ECOG performance status (*n* (%))				
– Score of 0	108 (71)	57 (74)	51 (68)	
– Score of 1	34 (22)	16 (21)	18 (24)	
– Score of 2	10 (7)	4 (5)	6 (8)	
Oropharyngeal cancer HPV status				
– Patients included (*n*)	51	32	19	
– Positive (*n* (%))	31 (61)	20 (63)	11 (58)	
– Negative (*n* (%))	15 (29)	10 (31)	5 (26)	
– Unknown (*n* (%))	5 (10)	2 (6)	3 (16)	
Tumour site (*n* (%))				
– Oropharynx	51 (34)	32 (42)	19 (25)	0.03*
– Oral cavity	49 (32)	19 (25)	30 (40)	0.04*
– Larynx	31 (20)	18 (23)	13 (17)	0.36
– Neck – unknown primary	10 (6)	4 (5)	6 (8)	0.49
– Other^†^	11 (7)	4 (5)	7 (9)	0.33
Disease stage (*n* (%))				
– I	33 (22)	8 (10)	25 (33)	<0.001*
– II	24 (16)	12 (16)	12 (16)	0.89
– III	24 (16)	12 (16)	12 (16)	0.89
– IV	69 (45)	45 (58)	24 (32)	<0.001*
– Unknown	2 (1)	0 (0)	2 (3)	
Primary treatment modality (*n* (%))				
– Surgery alone^‡^	47 (31)	7 (9)	40 (53)	<0.001*
– Radiotherapy**	105 (69)	70 (91)	35 (47)	<0.001*

*p < 0.05 by chi-square test, Fisher exact test. ^†^Salivary gland, nasopharynx, sinonasal (nose, sinuses, skull base), hypopharynx. ^‡^Open or endoscopic procedure. **Radiotherapy alone, or in combination with chemotherapy and/or surgery. ECOG = Eastern Cooperative Oncology Group; HPV = human papillomavirus

### Swallow dysfunction

**Fig. 2.**
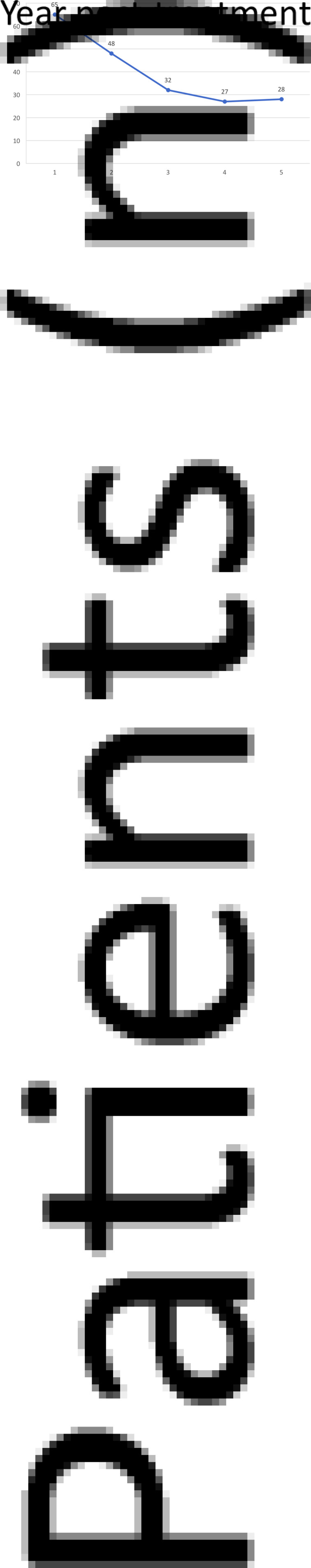
Total numbers of patients reporting symptoms of swallow dysfunction, as described in clinical letters, each year post-treatment.

Of the 152 included patients, 77 (51 per cent) were categorised into the swallow dysfunction group and 75 (49 per cent) into the normal swallow group. All patients in the swallow dysfunction group demonstrated evidence of swallowing problems at some point during the five-year follow-up period (Table 1). Figure 2 illustrates the number of patients reporting dysphagia in the swallow dysfunction group each year up to five years post-treatment. The majority of these patients (*n* = 65, 84 per cent) reported dysphagia in the first year. This rate fell in the subsequent two years, with 35–36 per cent of these patients still reporting symptoms in years four to five, indicating long-term swallow dysfunction.

Some patients were followed up for longer than five years, which is typically the point of discharge. Sixty-five patients (84 per cent) in the swallow dysfunction group were seen for up to six years, and 12 (16 per cent) were in their seventh year of follow up. The normal swallow group also contained patients followed up to six years (*n* = 58, 77 per cent) and seven years (*n* = 17, 23 per cent).

Evidence of newly occurring swallow dysfunction was documented in patients at up to five years post-treatment. Of the 28 patients who reported swallow dysfunction in their fifth year of follow up, 2 patients had not complained of symptoms prior to year four. One patient had no symptoms of swallow dysfunction in any previous years.

### Treatment modality and swallow dysfunction

A significantly higher percentage of patients who were treated for stage IV cancer (*p* < 0.001) experienced swallow dysfunction post-treatment, with the opposite being shown in stage I cancer patients (*p* < 0.001). Patients with oropharyngeal tumours were also more common (*p* = 0.03) in the swallow dysfunction group, whereas patients with oral cavity tumours were more likely (*p* = 0.04) to have normal swallow function.

A total of 105 patients underwent RT, either standalone or as part of combined treatment. All patients who underwent RT were treated with volumetric modulated arc therapy – a type of intensity-modulated RT. Seventy (67 per cent) of these patients showed evidence of swallow dysfunction, compared to seven patients (15 per cent) who only had a surgical procedure. Radiotherapy was significantly associated with swallow dysfunction (*p* < 0.001).

### Patient-reported quality of life outcomes

The MD Anderson Dysphagia Inventory was completed by 33 patients (43 per cent) in the swallow dysfunction group and by 23 patients (31 per cent) in the normal swallow group. There was a strong correlation (Pearson r = 0.77, *p* < 0.001) between MD Anderson Dysphagia Inventory global and composite scores. Patients in the normal swallow group had a significantly higher mean MD Anderson Dysphagia Inventory global score (*p* = 0.01) compared to the swallow dysfunction group (Table 3). Although patients in the normal swallow group had a higher mean composite score, there was no significant difference compared to the swallow dysfunction group. Similarly, patients who underwent surgery alone scored significantly higher (*p* = 0.03) in the MD Anderson Dysphagia Inventory global assessment compared to patients who had RT, but there was no significant difference in MD Anderson Dysphagia Inventory composite scores (Table 4).

**Table 3.** MDADI scores for swallow dysfunction and normal swallow groups

Questionnaire	Swallow dysfunction group (*n* = 33)	Normal swallow group (*n* = 23)	*P*-value
MDADI global score	49.1 ± 27.9	72.2 ± 34.0	0.01*
MDADI composite score	66.6 ± 16.7	75.1 ± 18.9	0.05
EQ-VAS	65.4 ± 26.3	65.4 ± 20.0	0.91

Data represent mean (± standard deviation) MD Anderson Dysphagia Inventory (MDADI) scores, unless indicated otherwise. *p < 0.05 by Mann–Whitney U test. EQ-VAS = EuroQol visual analogue scale

**Table 4.** MDADI scores for radiotherapy and surgery only groups

Questionnaire	Radiotherapy group (*n* = 40)	Surgery only group (*n* = 16)	*P*-value
MDADI global score	52.0 ± 28.6	75.0 ± 36.1	0.03*
MDADI composite score	67.6 ± 17.1	76.1 ± 19.3	0.06
EQ-VAS	66.7 ± 23.8	62.4 ± 23.8	0.64

Data represent mean (± standard deviation) MD Anderson Dysphagia Inventory (MDADI) scores, unless indicated otherwise. *p < 0.05 by Mann–Whitney U test. EQ-VAS = EuroQol visual analogue scale

**Fig. 3.**
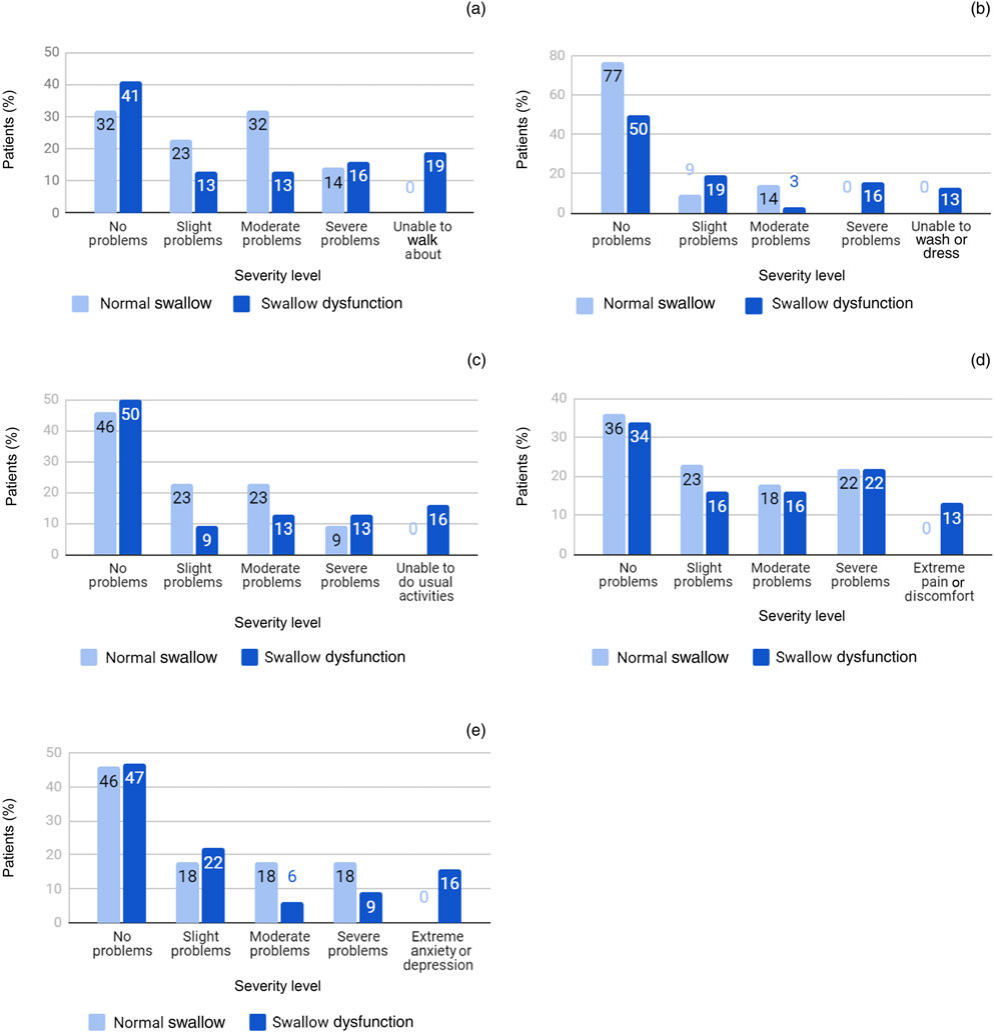
Proportion of responses for EuroQol five-dimensions five-levels (‘EQ-5D-5L’), by level of severity, for normal swallow and swallow dysfunction groups: (a) mobility, (b) self-care, (c) usual activities, (d) pain or discomfort, and (e) anxiety or depression.

**Fig. 4.**
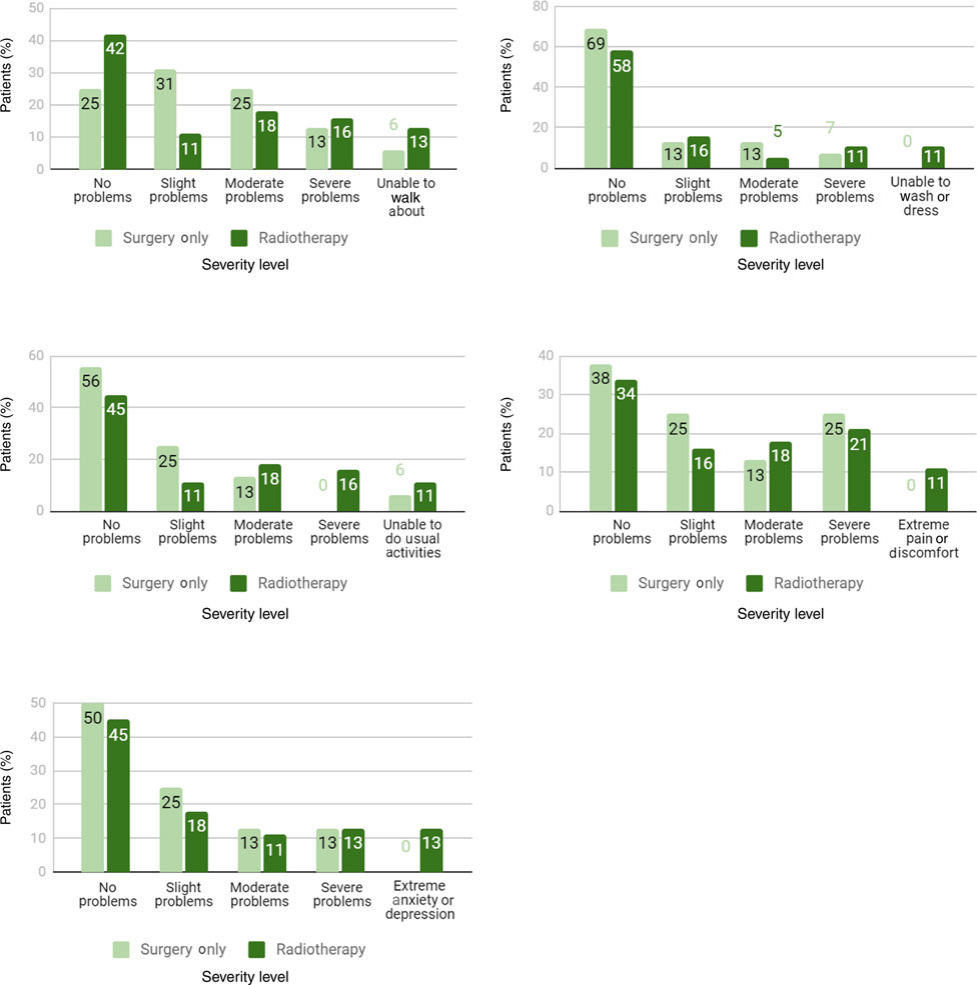
Proportion of responses for EuroQol five-dimensions five-levels (‘EQ-5D-5L’), by level of severity, for radiotherapy patients and surgery only patients: (a) mobility, (b) self-care, (c) usual activities, (d) pain or discomfort, and (e) anxiety or depression.

The EuroQol five-dimensions instrument was completed by 32 patients (42 per cent) in the swallow dysfunction group and by 22 patients (29 per cent) in the normal swallow group. More patients from the swallow dysfunction group and RT group reported the worst descriptor (extreme problems/unable to perform) across every dimension compared to the normal swallow group, and a similar result was found for patients who underwent surgery alone respectively, but these findings were not statistically significant (Figures 3 and 4).

The EuroQol VAS scores did not show a significant difference between swallow dysfunction and normal swallow groups, or between RT and surgery groups. The MD Anderson Dysphagia Inventory global score, which measures overall swallowing-related QoL, and the EuroQol VAS, which measures perceived overall health, showed a significant correlation at the 0.01 level (two-tailed).

## Discussion

These findings provide insight into the significance of long-term adverse swallowing effects associated with head and neck cancer treatment, occurring late into follow up. The results show that swallow dysfunction is a long-term problem for many surviving head and neck cancer patients, especially those treated with RT. Although research in this field is still relatively sparse, our findings are similar to those of earlier relevant studies from the UK^11^ and the USA.^12^

### Treatment modalities and associated adverse outcomes

With the growing population of long-term head and neck cancer survivors, understanding long-term post-treatment toxicities is important. Patients who underwent RT had significantly higher rates of post-treatment swallow dysfunction, compared to patients who underwent surgery alone. This will potentially become a greater problem as more patients with head and neck SCC are treated with RT and survive long-term.

Significant improvements in radiation techniques have come to the fore in recent years, such as the introduction of intensity-modulated RT. Volumetric modulated arc therapy offers greater treatment efficiency, a highly conformal radiation dose distribution and greater sparing of swallowing-related tissues, thereby improving swallowing outcomes compared to conventional RT.^16,17^ Additional benefits may be gained from the optimisation of intensity-modulated RT by oncologists for the sparing of swallowing structures.^16^

### Long-term dysphagia post-treatment

Dysphagia is a prevalent and debilitating long-term problem for many head and neck cancer survivors. Half of all included patients had some evidence of swallow dysfunction during the five years post-treatment. A study from Iowa, which examined risk factors for dysphagia, and the association between severity and survival, showed similar results, with 45.9 per cent of 407 patients identified as having dysphagia post-treatment.^9^ In addition, Hutcheson *et al*. reported a 45.3 per cent prevalence of dysphagia from Surveillance, Epidemiology, and End Results (‘SEER’) data analysis.^18^

The incidence of swallow dysfunction decreased in the five-year follow-up period, with the highest rate being in the first year. The long-term swallow dysfunction rate was 36 per cent for patients in the swallow dysfunction group, and 18 per cent for all included patients. Newly occurring swallow dysfunction in the later years of follow up (after year four) was documented in three patients. Ward *et al*. identified severe late dysphagia in almost one-third of their patients, which persisted at the five-year follow up.^19^ Late-onset dysphagia was also described, whereby 3 (18 per cent) of 22 patients with severe late dysphagia had their first occurrence after five years post-treatment.^19^ Swallow dysfunction following head and neck cancer treatment may not only be long-term, but could also present much later in a subset of patients. Patients therefore require close monitoring, and future studies should explore the timing and course of swallowing outcomes after head and neck cancer treatment.

### Post-treatment quality of life

Our results showed a greater mean MD Anderson Dysphagia Inventory global score in the normal swallow group, indicating better overall swallowing-related QoL in patients with no evidence of swallow dysfunction. However, there was no significant difference in terms of MD Anderson Dysphagia Inventory composite or EuroQol five-dimensions instrument scores compared to the swallow dysfunction group, despite there being a correlation between EuroQol VAS (measuring perceived overall health) and MD Anderson Dysphagia Inventory global scores. This could be the result of patient factors not addressed in this study, such as coping mechanisms and depression, which can impact QoL and patient-perceived functioning.^20,21^

A 2011 study utilising the EuroQol five-dimensions instrument to investigate late treatment toxicity on QoL in 396 head and neck cancer RT patients showed that dysphagia and xerostomia had a significant negative impact on QoL after at least six months post-treatment, with dysphagia having the greater impact.^22^ The MD Anderson Dysphagia Inventory global scores in the present study are in line with this finding. Reports relating to QoL outcomes at five years post-treatment for head and neck cancer are difficult to collect because of attrition in subject numbers. The present study contributes to the limited body of evidence thus far.

Global and composite MD Anderson Dysphagia Inventory scores reflected patients’ perception that dysphagia negatively impacted their QoL in a retrospective study that explored patient-reported versus physiological swallowing outcomes in 30 patients who underwent chemoradiotherapy.^23^ However, physiological swallow dysfunction outcomes did not correlate with MD Anderson Dysphagia Inventory scores, similar to our findings. A greater decline in physiological dysphagia outcomes over time suggested there may be a decrease in patient awareness of dysphagia in the years following chemoradiotherapy.^23^ However, the small cohort of patients in the study was a limitation.

### Limitations

This study is a descriptive retrospective case series, which limits the reliability of results. Assessing for swallow dysfunction criteria retrospectively relies on accurate and thorough documentation of a patient's reported symptoms and their clinician's investigation results. The variation in quality and non-standardised nature of this documentation is likely to result in an underestimation regarding swallow dysfunction prevalence. A prospective long-term follow-up study may provide a more robust and complete dataset of the morbidity that these patients experience.

- Head and neck cancer and its treatment can result in short- and long-term morbidities, affecting quality of life
- Swallow dysfunction is a demanding late effect of head and neck cancer treatment that may develop or progress years post-treatment
- Half of head and neck cancer survivors showed evidence of swallow dysfunction at some point post-treatment
- Long-term swallow dysfunction was seen in 18 per cent of all included patients at five years’ follow up
- Radiotherapy was significantly associated with swallow dysfunction compared to other treatment modalities

Another limitation was the inability to calculate the true prevalence of adverse outcomes in these patients. The QoL questionnaires were only completed by a small sample in each group, reflecting our restricted ability to contact these patients during the pandemic. An absence of baseline scores precluded any pre-treatment comparison, thereby limiting any conclusion drawn from the results. We do acknowledge that this information was gathered a relatively long time after their treatment, and not all patients are in regular contact with hospital services. Despite this, there are very little long-term data on patients at extended follow-up times, and the findings of this sample contribute to the current literature. Furthermore, some patients had to be excluded because of missing information on the electronic record.

### Areas for future research

New trials are emerging that investigate the avoidance of head and neck cancer toxicity without compromising patient survival. Findings in the coming years will shape our understanding of how to better prevent swallow dysfunction in head and neck cancer survivors.^24^ Areas of future research include predictors of severe fibrosis and variables for aspiration pneumonia, and preventative measures for these. Furthermore, reliable screening tools to detect long-term aspiration and dysphagia are required.^11^

## Conclusion

Side-effects of head and neck cancer treatments, including dysphagia and other long-term functional outcomes, are common and have devastating effects. Half of head and neck cancer survivors showed evidence of swallow dysfunction at some point post-treatment. Long-term swallow dysfunction was apparent in this study. Evidence of swallow dysfunction was present in 36 per cent of patients during the first five years post-treatment, and in 18 per cent of all patients at five years’ follow up. Radiotherapy was significantly associated with swallow dysfunction compared to other treatment modalities. The QoL questionnaire scores reflected an overall patient-perceived negative impact on QoL in those with evidence of swallow dysfunction compared to those without. Future studies should explore the timing and course of swallowing outcomes after head and neck cancer treatment, as late adverse effects can be unpredictable and difficult to manage.
